# Quality audits of radiotherapy centres in Latin America: a pilot experience of the International Atomic Energy Agency

**DOI:** 10.1186/s13014-015-0476-7

**Published:** 2015-08-14

**Authors:** Eduardo Rosenblatt, Eduardo Zubizarreta, Joanna Izewska, Sergio Binia, Fernando Garcia-Yip, Pablo Jimenez

**Affiliations:** Applied Radiation Biology and Radiotherapy Section, Division of Human Health, International Atomic Energy Agency, Wagramerstrasse 5, PO Box 100, A-1400 Vienna, Austria; Dosimetry and Medical Radiation Physics Section, Division of Human Health, International Atomic Energy Agency, Vienna, Austria; Radiotherapy Department, Fundación Escuela de Medicina Nuclear (FUESMEN), Mendoza, Argentina; Radiotherapy Department, Instituto Nacional de Oncología y Radiobiología (INOR), Havana, Cuba; Unit of Medicines and Health Technologies; Pan American Health Organization (PAHO); World Health Organization (WHO), Washington, USA

## Abstract

**Background:**

In Latin America radiotherapy quality varies significantly among hospitals, where highly equipped academic centers coexist with others not meeting minimal requirements. In 2007, the International Atomic Energy Agency published guidelines for auditing radiotherapy centers, known as the “Quality Assurance Team for Radiation Oncology” (QUATRO) audits. The present report summarizes a pilot experience with QUATRO audits to 12 radiotherapy centres.

**Methods:**

The findings from QUATRO audits conducted in 12 radiotherapy centres in Latin America between 2008 and 2013 were analysed. Events representing weaknesses or gaps in the process of radiotherapy were recorded. Relevant data for estimating human and technological needs of visited centres were processed. The main difficulties and strengths faced by institutions were also documented.

**Results:**

All 12 radiotherapy centres were successfully audited following the QUATRO method. IAEA provided a dosimetry kit for quality control. Forty percent of audited institutions were immersed in a health system that did not recognize cancer as a public health priority problem. With few exceptions, local training programs for physicists and technologists were scarce and research was not an activity of interest among physicians. Centres were provided with sufficient staff to meet the local demand, both in the case of radiation oncologists, physicists and radiation therapists. Three centres lacking the minimum infrastructure were identified. Three institutions did not perform gynaecological brachytherapy, and one installation delivered around 900 teletherapy treatments annually without simulation, planning or dosimetry equipment for that purpose. Recommendations to centres were classified as related to personnel, infrastructure, processes and institutional organizational aspects. Many recommendations warned governments about the evident need for allocating more budgetary resources to radiotherapy. Most recommendations pointed out different aspects related to strengthen human resources training and technological support to the audited centres. Scheduled follow-up visits were also stressed.

**Conclusion:**

The QUATRO audits proved to be a valuable tool for identifying weaknesses in infrastructure, human resources and procedures in radiotherapy centres. Follow-up visits conducted by the IAEA or by regional or local organizations are necessary in order to evaluate outcomes and sustainability of implemented recommendations.

## Introduction

Latin America includes countries in Central America, South America, the Caribbean and México, covering nineteen countries and about 600 million inhabitants [1].

There is notable inequity in access to health services within the region. The scenario is characterized by significant heterogeneity in infrastructure and supply of services both within and between countries [2, 3]. This is also the case for oncology in general and radiation oncology in particular [3].

The cancer incidence in Central America is of 134 cases per 100,000 people per year, and 190 new cases is the number for South America [4]. The availability of megavoltage therapy machines varies from less than 1 to more than 4 units per million people between the least and most equipped countries respectively. The needs are covered in a 58─75 %, depending on the country [5]. Most professionals specialized in cancer and its treatment are concentrated in the largest cities. The increasing migration of people from rural areas to cities has resulted in a predominance of urban health services deepening the inequity which affects the most vulnerable sectors [3].

The quality of oncology services also varies significantly between hospitals, and highly equipped academic centres coexist with facilities not meeting the minimal requirements of a basic clinic [6]. Collected data and recommendations related to these aspects constitute the body of this publication.

## Background and purpose

Analysis of indicators obtained through surveys and audits is a widely used methodology to measure performance of health services [7, 8], including radiation oncology providers [9].

The Division of Human Health of the International Atomic Energy Agency (IAEA) published guidelines for audits of radiotherapy centres in 2007, known as QUATRO (“Quality Assurance Team in Radiation Oncology”) audits [10]. The publication contains checklists that auditors ought to complete, the analysis of which allows measurement of the degree of development of the audited institution. The composition of the on-site visit depends on the scope, level of development and expected content of the audit visit, but in all cases involved a radiation oncologist, a radiotherapy physicist and a radiation therapist (RTT). A fourth member with special competencies such as a radiation protection officer was engaged from the visited country. Data collected by the auditors normally include observations concerning buildings, human resources, treatment and dosimetry equipment, comprehensive patient care, adherence to standards of radiation protection and establishment of quality assurance programmes, education and research [11–14].

Given that QUATRO audits are voluntary, only centres that desire and formally request participation were audited. The final aim of a QUATRO audit is a draft of recommendations to the audited centre, IAEA and governmental authorities of the country where the institution is located in order to optimize the quality of clinical care provided to patients who receive radiotherapy.

Audit reports are confidential. The intention is never to disseminate findings relating to a particular centre or to investigate accidents or alleged punishable actions. This publication summarizes the results of 12 such QUATRO audits in Latin America.

## Materials and methods

The reports from QUATRO audits conducted in 12 radiotherapy centres in Latin America between 2008 and 2013 were analysed. The sample of centres to be audited was selected during an IAEA regional project coordinators’ meeting in 2007 during which country representatives selected radiotherapy centres of diverse level of development across the region. The purpose was to validate the QUATRO method in Latin America and further encourage countries to develop their own national audit systems based on this method. As part of a joint project between the IAEA and the Pan American Health Organization (PAHO), Regional Office of the World Health Organization for the Americas, to support the Action Plan on Cancer Control for Central America and the Dominican Republic, PAHO facilitated the circulation of the IAEA dosimetry kit and provided an additional expert to join the IAEA teams for the audits held in those countries.

Drawbacks for the actual audit implementation were recorded. Relevant data for estimating human and technological needs of the centres audited were processed. In the first case, estimates were made by using a tool developed by the IAEA for calculating ‘necessary personnel’ based on existing staff [15]. The theoretical calculation of ‘necessary machines’ was based on IAEA recommendations [6]. The main difficulties faced by institutions were documented as well as their strengths.

A coding scheme was developed to categorize recommendations that auditors made to the audited centres. The authors discussed, agreed and assigned a code to each recommendation. The codes were loaded into an internally designed Excel spreadsheet to be summed and the frequency of each recommendation was obtained. Recommendations to governments and to IAEA were manually processed.

The analysis of reports and causality were conducted by a working group of the Division of Human Health of the IAEA. Confidentiality was maintained throughout the process.

## Results and discussion

### Audited centres

Twelve radiotherapy services located in 10 Latin American countries (4 in South America and 6 in Central America) were audited from 2008 to 2013. Due to the selection method described above, the sample is small compared to the total number of facilities existing in the region; 649 radiotherapy centres as of March 2015 [5, 16–18]. Hence this report should not be construed as representative of the overall situation of Latin America.

Two institutions were categorized as ‘not for profit’ private hospitals while all others belonged to the public health system of the country involved.

It was noted that most audits had been requested voluntarily by health system personnel with the agreement and consent of heads of departments. A single service was exceptional, where the staff advised that the audit had been imposed by hospital authorities. However staff members in this case, as in all the others, were keen to assist and collaborate with the auditors to achieve the objectives of the mission.

### Drawbacks for implementing the audits

Problems regarding the equipment that the IAEA sent for quality control of irradiation machines and clinical dosimetry devices (dosimetry kit) were reported in four instances. This happened in the first missions due to protracted retention of the IAEA dosimetry kit in local customs. To overcome this problem, in subsequent audits the dosimetry kit was sent to the countries through PAHO official channels several weeks before the visit. In the first three visits, measurements were not possible due to this obstacle. In an additional one, measurements were conducted using dosimetry equipment brought by one of the auditors as per IAEA request. In the others, measurements were carried out successfully.

One audited centre had no clinical dosimetry equipment and thus only quality control of the single operating therapy machine (^60^Co unit) was possible.

### The setting of the audited centres. Shortcomings and difficulties

Forty percent of audited institutions belonged to health systems that did not prioritise cancer as a public health issue, nor recognised the different disciplines that make up a radiotherapy team, in particular medical physics.

With few exceptions, local training programs for physicists and technologists were scarce and research was not an activity of interest among physicians.

Some degree of demotivation of workers was evident in 30 % of centres, and a near constant absence of quality management programmes was reported. These factors impacted negatively on the performance of services, as evidenced by the long lists of patients waiting to receive care in more than half of the audited centres.

Given that cervical cancer is the malignancy with the second highest incidence and mortality among females in the region [4, 19, 20], it is a striking and disturbing fact that three of the audited centres do not perform gynaecological brachytherapy procedures as part of the curative treatment of the disease. Lack of equipment, referral of patients to other facilities that provide the practice and/or lack of paying capacity of patients to afford the costs were the commonest reasons.

Although lack of equity in the provision of services depending on the economics of patients was not registered as a frequent event, cases of inequitable use of technology were identified, including external radiotherapy with ^60^Co vs linear accelerator, restriction of gynaecological treatments and omissions in computed tomography simulation (planning in the treatment machine vs CT simulation).

Radiation protection was frequently not considered an important issue; its management was deficient and international recommendations [21, 22] were not met in half of the audited installations.

### Highlighted virtues of audited centres

Only a single instance of sloth and poor cooperation with auditors was reported. Most reports highlighted an excellent disposition and collaborative attitude of institutional staff at all levels of the hierarchy.

One department was designated a ‘centre of competence’ by the audit team, which also stressed that with the support of higher authorities it could become a ‘centre of excellence’ [6].

Those institutions that offered formal training programmes such as residency programmes for physicians and physicists and continuing education activities were categorized as ‘academic centres’ [23, 24]. Three centers in South America and one in Central America received this rating.

### Human resources and staff needs

Contrary to most publications concerning radiation oncology services in Latin America and the Caribbean [5, 16, 17] where needs have been calculated based on population of the respective countries [1, 3, 25], the centres audited by the IAEA generally had sufficient staff numbers to meet requirements both in the case of radiation oncologists and supporting staff (physicists and radiation therapists) [26–30]. Despite large numerical differences among departments, when each one is analysed separately, it is evident that the estimated minimum number of workers required is exceeded by existing ones in most cases. This was true for technical staff in eight (67 %) of the twelve centres audited and physicists and radiation oncologists in seven (58 %) (Table 1).

**Table 1:** Staff present and necessary per audited centre

Centers	Radiation oncologists		Medical physicists		Radiotherapy technologists	
	Existing^(a)^	Needed^(b)^	Existing^(a)^	Needed^(b)^	Existing^(a)^	Needed^(b)^
I	9.6	11.3	6.7	5.8	15.8	26.3
II	5.7	5.3	2.4	2.9	6	12.5
III	2.9	1.6	2.2	2.2	8.3	4.4
IV	5.4	11.9	3.9	7.6	16.3	19
V	9	5.6	7.6	4.1	18	11.1
VI	1.9	0.6	0.8	0.9	4	2.4
VII	3.8	4.4	2.9	2.1	8	10.7
VIII	4.8	3.9	6.7	3	15	10.5
IX	0.9	1.7	1.9	1.2	8.2	6.7
X	14.3	10.7	6.7	7.5	27	19.3
XI	1.4	4.1	2.9	1.3	11.3	4.5
XII	4.3	1.7	3.2	1.4	4.5	3.4
Sub-region	Radiation oncologist		Medical physicist		Radiotherapy technologist	
	Existing^(a)^	Needed^(b)^	Existing^(a)^	Needed^(b)^	Existing^(a)^	Needed^(b)^
Central America	~37	~34	~34	~21	~89	~78
South America	~27	~29	~14	~19	~53	~53

^(a)^Full time equivalent workers

^(b)^Calculations based on 8 working hours per day and 5 days per week

Interestingly, estimates indicated that one of the academic centres required double its existing physical and medical staff. This is because calculations directly increase the theoretical staff requirements depending on the technological resources of the department. Also, residents do not factor in calculations of personnel needs, while in reality the more advanced trainees in this centre undertake a significant workload. When the numbers of this particular centre are merged with those of other centres for a sub-regional estimation, its influence on the result indicates a slight lack of radiation oncologists and physicists in audited centres in South America.

The same effect but in the opposite direction, occurs when centres with major infrastructure ‘compensate’ for centers with more modest ones. Merging data into sub-regions makes differences between them ‘disappear’. For example, two departments were found where radiation oncologists were treating more than double the recommended number of patients with teletherapy (Table 2) [26–30], but this is not perceptible when presenting the results by sub-region (Table 1).

**Table 2:** Workload of radiation oncologists and medical physicists

Centers	EBRT courses per professional (FTE)	
	Radiation oncologists	Medical physicists
I	201	288
II	118	280
III	172	227
IV	424	587
V	178	211
VI	183	435
VII	294	386
VIII	314	225
IX	1000	474
X	151	322
XI	576	278
XII	115	155

### Facilities and equipment

Some centres lacking the minimum infrastructure recommended by the IAEA and other organizations [26–30] were identified. Three institutions did not perform gynaecological brachytherapy as previously mentioned. In addition, one installation delivered around 900 teletherapy treatments annually without simulation, planning or dosimetry equipment for that purpose (Table 3).

**Table 3:** Equipment and services per audited centre and per sub-region

Centers	Simulation^(a)^	TPS^(b)^	EBRT machines^(c)^		BT services^(d)^		Dosimetry^(e)^
I	1	2	5		3		Yes
II	2	1	2		1		Yes
III	1	2	2		1		Yes
IV	2	8	5		2		Yes
V	2	4	5		1		Yes
VI	1	2	2		0		No
VII	1	0	2		1		Yes
VIII	2	3	4		2		Yes
IX	0	0	1		0		No
X	2	6	6		1		Yes
XI	2	1	1		1		Some
XII	1	1	1		0		Yes
Sub-region	Simulation^(a)^	TPS^(b)^	EBRT machines^(c)^		BT services^(d)^		Dosimetry^(e)^
			Existing	Needed^(f)^	Existing	Needed^(g)^	
Central America	10	13	21	18	9	11	Most yes
South America	7	17	15	11	4	5	Most yes

^(a)^Radioscopic and computed tomography simulators

^(b)^2D and 3D treatment planning systems

^(c)^Orthovoltage irradiators, ^60^Co units and linear accelerators

^(d)^Low dose rate and high dose rate brachytherapy facilities

^(e)^Clinical dosimetry

^(f)^Calculations based on 1 machine needed each 500 treatment courses [5]

^(g)^Calculations based on 1 facility needed each 200 treatment courses [5]

The technological capabilities were very heterogeneous among audited centres but as in the case of staff, differences were barely perceptible when data were collated (Table 3).

### Recommendations from QUATRO audit teams

#### To audited centres

The recommendations to the audited centres were classified into personnel, infrastructure, processes and institutional organizational aspects.

##### Related to personnel

**Fig. 1:**
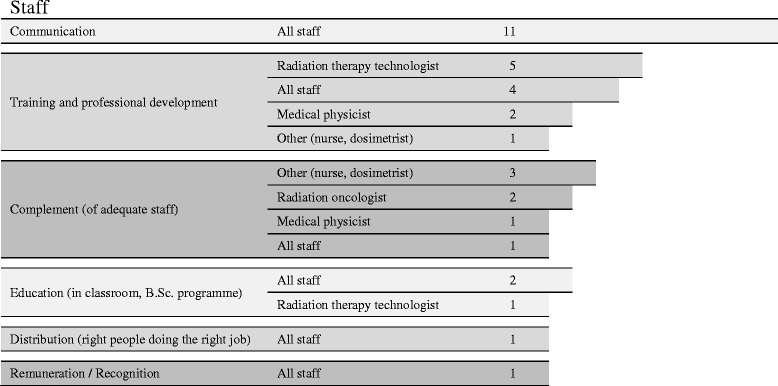
Recommendations to centres regarding staff. Numbers depict how many times a recommendation was found in the QUATRO reports. For example: the first row means that the different audit teams have recommended 11 times that all staff members should improve internal and external communications of audited centres

Improving communication among internal staff, among radiation oncologists with practitioners from other hospital departments and between the audited institution and other organizations was the most frequent recommendation in this category. Improving training and encouraging professional development to workers in general, and technologists in particular, was also a common recommendation (Fig. 1).

##### Related to infrastructure

Infrastructure includes all elements and technological resources that enable proper treatment planning and delivery. Furthermore it includes devices for clinical dosimetry and quality control of machines, computer platforms including network connections, and building aspects such as bunkers, changing rooms, meeting rooms, access pathways (wheelchairs, stretchers) and associated services.

**Fig. 2:**
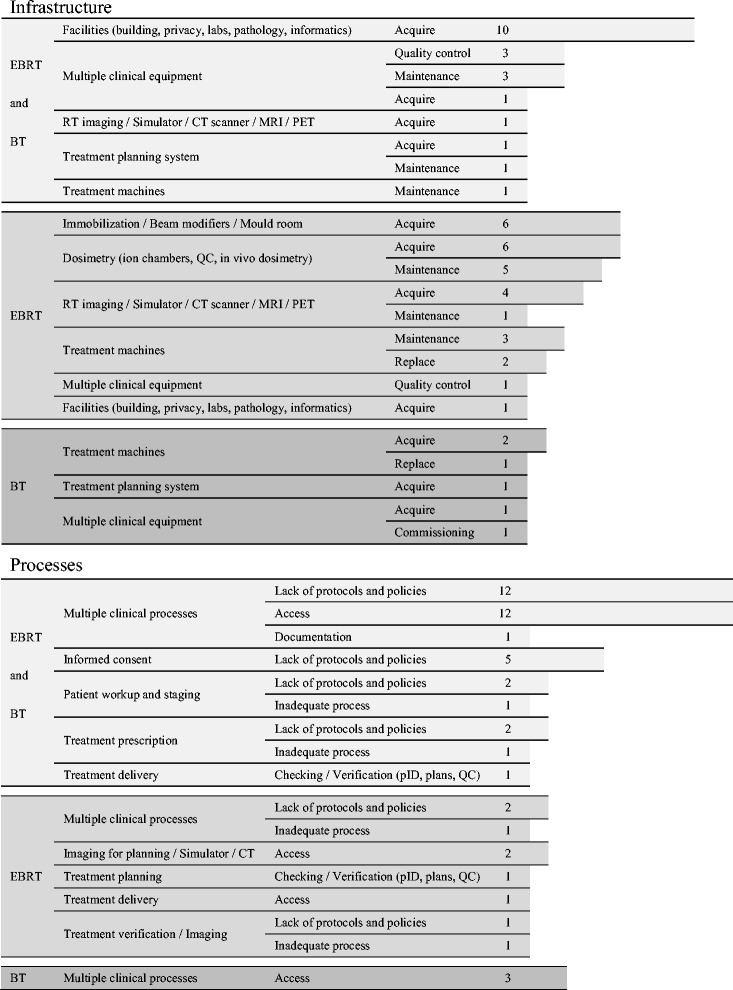
Recommendations to radiotherapy centres regarding infrastructure and processes. Numbers depict how many times a recommendation was found in the QUATRO reports. For example: the first row on ‘Infrastructure’ means that the different audit teams have recommended 10 times that some equipment should be acquired to improve both teletherapy and/or brachytherapy services; the first row of ‘Processes’ means that the different audit teams have recommended 12 times that protocols and policies involving clinical processes should be developed and established in order to improve both teletherapy and brachytherapy services

The recommendations addressed topics concerning teletherapy and brachytherapy infrastructure together and each of the modalities separately (Fig. 2).

Installing, upgrading and expanding of computing resources stand out among the recommendations along with some aspects related to patient privacy. Acquiring devices for patient immobilization and clinical dosimetry as well as optimizing maintenance (preventive and corrective) of treatment machines were the commonest recommendations for teletherapy. All those centres without brachytherapy or with obsolete brachytherapy facilities were advised to install, upgrade and modernize this modality in the short term.

##### Relative to processes

As in the previous section, recommendations were divided into those concerning processes of external beam radiotherapy and brachytherapy alike, those aimed exclusively at teletherapy processes and the ones developed based on the findings of the auditors in brachytherapy services.

Both modalities, together and individually, did not adhere to protocols and institutional policies in most of the audited centres. Consequently the most frequent recommendation in this category was linked to these processes. Other very frequent recommendations highlighted the inadequate access of patients to multiple processes within institutions causing unacceptable waiting lists (Fig. 2). Waiting lists delayed patient access to the general process of radiotherapy as well as to each one of its individual components.

##### Related to organizational aspects

**Fig. 3:**
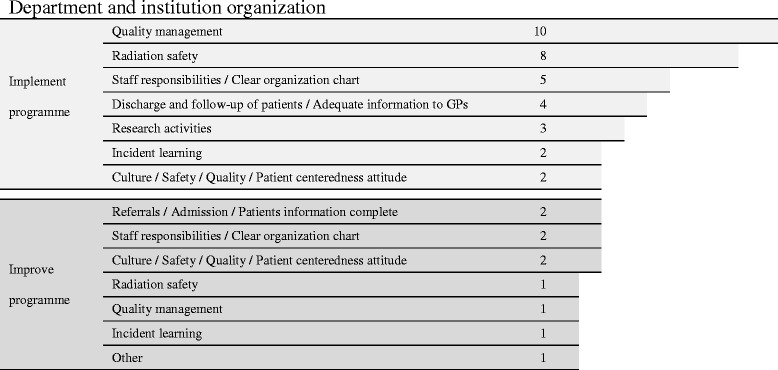
Recommendations to radiotherapy centres regarding department and institutional organization. Numbers depict how many times a recommendation was found in the QUATRO reports. For example: the first row means that the different audit teams have recommended 10 times that a quality management programme should be implemented in order to define, optimize and reach the institutional objectives, as well as to promote satisfaction to all internal and external users

Here recommendations were limited to two terms, implementing non-existing programmes and improving those programmes that run incompletely or improperly. Quality management, radiation safety, allocation of responsibilities, follow up of patients and clinical research activities were the main programmes which most departments were recommended to implement or optimize (Fig. 3). The change of attitude of organizations focusing more on patients’ needs was also a frequent recommendation.

Radiotherapy centres which had been affected by radiation overexposure events in the past [31, 32] were advised to implement comprehensive institutional programmes to allow them to resume their activities with the confidence and fluency which the specialty requires.

#### To governments

**Fig. 4:**
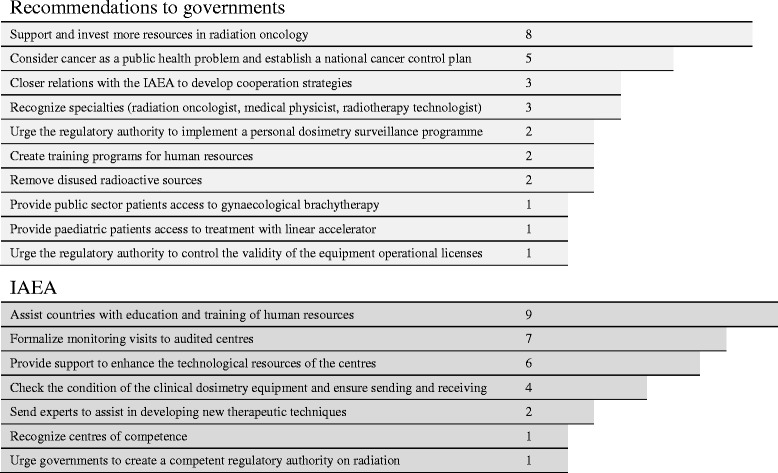
Recommendations to governments and to IAEA

Many recommendations advised governments on the evident need for allocating more budgetary resources to strengthen radiotherapy services (Fig. 4). Furthermore, the audit teams emphasized that neoplastic diseases must be considered a priority public health issue and that countries should implement comprehensive cancer control programs and ensure their sustainability.

#### To IAEA

The majority of recommendations alluded to different strategies to strengthen collaboration in human resource training and technological support to audited centres. Scheduled monitoring and follow-up visits were also stressed (Fig. 4). One of the audited centres received a follow-up visit 5 years following the original one. The report of this follow-up visits reflects a partial implementation of the recommendations issued in the first audit.

## Conclusions

Quality audits following the QUATRO method were a valuable tool for identifying weaknesses that radiotherapy centres had regarding staff, infrastructure, processes and institutional organization. The recommendations drafted by the auditors provide guidance to improve levels of operation and service delivery in evaluated departments. Follow-up visits conducted by the IAEA or by regional or local organizations are necessary in order to evaluate the outcomes and sustainability of implemented changes. Countries with a large number of radiotherapy centres could adopt the QUATRO methodology to develop their own national audit systems as part of their national quality programmes in radiotherapy.

## Abbreviations

IAEA: International Atomic Energy Agency
PAHO: Pan American Health Organization
QUATRO: Quality Assurance Team in Radiation Oncology
B.Sc.: Bachelor of Science
EBRT: External beam radiotherapy
BT: Brachytherapy
RT: Radiotherapy
CT: Computed tomography
MRI: Magnetic resonance imaging
PET: Positron emission tomography
QC: Quality control
pID: Identification of the patient
GP: General practitioner
FTE: Full time equivalent
